# Dog ownership supports the maintenance of physical activity during poor weather in older English adults: cross-sectional results from the EPIC Norfolk cohort

**DOI:** 10.1136/jech-2017-208987

**Published:** 2017-07-24

**Authors:** Yu-Tzu Wu, Robert Luben, Andy Jones

**Affiliations:** 1 Department of Population Health and Primary Care, Norwich Medical School, University of East Anglia, Norwich, UK; 2 UKCRC Centre for Diet and Activity Research (CEDAR), Cambridge Institute of Public Health, Cambridge, UK; 3 Department of Public Health and Primary Care, Cambridge Institute of Public Health, University of Cambridge, Cambridge, UK

**Keywords:** physical activity, elderly, epidemiology

## Abstract

**Background:**

Dog ownership has been suggested to encourage physical activity in older adults and may enhance resilience to poor environmental conditions. This study investigates the role of dog ownership and walking as a means of supporting the maintenance of physical activity in older adults during periods of inclement weather.

**Methods:**

The analysis used data from the European Prospective Investigation into Cancer and Nutrition Norfolk cohort. Daily physical activity (counts per minute) and minutes of sedentary behaviour were measured using accelerometers over 7 days. Three types of environmental conditions, day length, precipitation and maximum temperature, were date matched with daily physical activity. A multilevel first-order autoregressive time-series model quantified the moderating effect of self-reported dog ownership and walking on the association between physical activity and weather factors.

**Results:**

Among the 3123 participants, 18% reported having a dog in their households and two-thirds of dog owners walked their dogs at least once a day. Regular dog walkers were more active and less sedentary on days with the poorest conditions than non-dog owners were on the days with the best conditions. In days with the worst conditions, those who walked their dogs had 20% higher activity levels than non-dog owners and spent 30 min/day less sedentary.

**Conclusion:**

Those who walked dogs were consistently more physically active than those who did not regardless of environmental conditions. These large differences suggest that dog walking, where appropriate, can be a component of interventions to support physical activity in older adults.

## Introduction

High levels of physical inactivity in older adults present a challenge to active ageing. In the UK, for example, it is estimated that less than 50% of older adults meet recommended physical activity levels of at least 150 minutes of moderate intensity activity per week.^1^ Physical activity promotion in primary care has been a major focus of research in recent years. Yet despite promise, particularly in older populations who more regularly visit their doctor, there has been limited evidence of substantial success. A systematic review of the effectiveness of physical activity promotion interventions in primary care published in The BMJ, for example, found evidence of effects at 12 months yet these were typically only small in magnitude.^2^ There is the need to identify factors that may increase the likelihood of any improved physical activity habits being maintained.

In the absence of evidence on the efficacy of individual interventions, some research has focused on modifying physical and social environments to reduce potential barriers to active ageing.^3 4^ However, some environmental conditions, such as poor weather and short day length, are beyond the direct control of planners yet have been related to decreased levels of physical activity in older adults.^5–7^ In such cases, the goal of interventions may be to enhance individual resilience to these poor conditions. A growing body of evidence suggests that dog ownership is associated with higher levels of physical activity in adults in all ages.^8–10^

Dog walking has been suggested to be a means of physical activity promotion in older adults.^10 11^ Evidence from observational studies shows a positive relationship between dog walking and physical activity in older people across different countries and regions, including USA, Canada and UK.^10–16^ For example, using the Health and Retirement Study, a nationwide cohort of older adults aged 50 or above in the USA, reported that dog walking was associated with higher frequency of self-reported physical activity.^11^ A small number of intervention studies have also suggested the beneficial effect of dog walking on leisure-time walking and adherence to physical activity programmes.^17 18^ A pilot randomised control trial provided educational materials to dog owners who did not walk their dogs regularly and reported increased walking time in the intervention group at 12-week follow-up.^17^ The other study used therapy dogs as an intervention in a walking programme and suggested a positive effect on adherence rates.^18^

Qualitative research suggests that dog walking may motivate older people to overcome poor weather conditions and promote regular outdoor activity.^19^ Although several environmental factors such as security, quality and sense of community^13 20 21^ have been related to dog walking behaviour, the potential for this to lead to maintenance of physical activity levels in poor weather conditions has not been well explored. Two quantitative studies have investigated the potential effect of dog ownership on seasonal differences in physical activity in adults of all ages.^22 23^ Lail *et al* 22 measured self-reported neighbourhood-based walking in summer and winter among 428 adults in Calgary, Canada, showing dog owners were most likely to report recreational walking in both seasons.^22^ The other study, also conducted in Canada, directly observed activities undertaken in six public parks over 12-week period and recorded visitors’ types of physical activity and the presence of dogs.^23^ The findings suggested that dog owners were more likely continue to visit parks in inclement weather.

Although the existing studies have provided some evidence on the potential for dog ownership to enhance resilience to poor environmental conditions, none specifically focused on older adults, a population with high health needs but who might be especially sensitive to poor environmental conditions.^24^ Studies also mainly focused on seasonal differences and did not objectively measure activity levels and daily weather conditions, which might lead to problems such as residual confounding with unmeasured influences. To address these issues, this study explores the effect of dog ownerships on the association between physical activity, sedentary behaviour and environmental conditions (day length, precipitation and temperature) using a large cohort of older adults in England.

## Methods

### Study population

This study uses data from the European Prospective Investigation into Cancer and Nutrition (EPIC) Norfolk study, one of population-based cohorts from the 10-county EPIC collaboration. The cohort was originally assembled to examine the associations between diet and cancer but has since been expanded to investigate major determinants of chronic disease, disability and death in middle and later life.^25^

Details of sampling and recruitment have been described elsewhere.^26^ Briefly, EPIC Norfolk recruited over 25 000 community-dwelling participants aged 40–79 between 1993 and 1997 from primary care across the county of Norfolk, a predominantly rural country of approximately 2000 square miles situated on the east coast of England. The climate of this area is relatively benign with a summer average maximum daytime temperature of 22°C and a winter night-time average minimum of 1°C. Between September 2006 and December 2011, as part of the 3rd Health Check (3HC), a sample of participants were asked to wear an accelerometer for a 7-day period as well as complete a questionnaire that, among other things, requested information on dog ownership and walking. The 3123 individuals who did this and provided valid data for the basis of the analysis presented here.

### Measure of physical activity

Physical activity was measured using a commercial accelerometer (Actigraph GT1M, Florida, USA), which was set to a 5 s epoch. The EPIC Norfolk participants attending the 3HC were invited to wear the accelerometer to measure their daily physical activity. Those who agreed to do so were instructed to wear the equipment during waking hours for continuous 7-day period. For the purpose of this analysis, valid days were defined as those with evidence that the accelerometer was worn for at least 10 hours.

For each participant, a summary of physical activity and sedentary time was computed for each valid day the Actigraph was worn. Daily counts per minute, a summarised indicator of daily activity level, were calculated using the total daily counts as recorded by the Actigraph divided by device total wear minutes. Sedentary behaviour was defined as valid periods of Actigraph wear where the device recorded under 100 cpm.^27^

### Dog ownership and walking

Dog owners were identified using the question ‘Does your household have a dog?’ in the questionnaire. Dog walking habits were measured based on the question ‘How often do you walk the dog?’ with four possible response options: never, sometimes, once a day and more than once a day. Dog owners reporting walking at least once a day were considered to have regular dog walking habits. Dog walking status was consequently classified as those who regularly walk their dogs (once a day), dog owners who did not frequently walk their dogs (<once a day) and non-dog owners.

### Environmental conditions: day length and weather

Meteorological information was obtained from the Marham weather station in Norfolk, England, the closest UK Meteorological Office station to the cohort. Marham is located 50 km from Norwich, the largest urban centre in the study area. Hourly measurements of temperature and precipitation were obtained for each day for which physical activity data were available. These were used to calculate daily cumulative precipitation (mm) between 06:00 and 22:00 and identify the maximum daytime temperature (Celsius). In addition, day length (hours) was computed based on an algorithm that used latitude.^28^

### Covariates

Demographic information on participant age, sex and education was included in the analyses. Education was divided into four levels: less than O level, O level (age 14–16), A level (age 16–18) and university degree or equivalent. Since poor health status has been associated with a lower level of physical activity,^29^ health status was measured by a self-reported question ‘How would you rate your general health?’. Those reporting ‘excellent’, ‘very good’ and ‘good’ health were categorised into one group and those reporting ‘fair’ and ‘poor’ were in another group.

### Analysis

As the data set comprised was a time series, multilevel first-order autoregressive models were fitted to take into account the repeated measure nature of the physical activity data and the autocorrelated structure of daily weather observations.^30^ A two-level structure was used of daily records (level 1) nested within individuals (level 2). Two sets of models were fitted; one with daily accelerometer counts per minute, a continuous measure of physical activity, as the outcome variable and one with time spent sedentary. Based on the statistical distributions of the weather data, maximum daytime temperature and day length were categorised into quartiles. Since many days had no precipitation (54%), dry days without any recorded rain were grouped into one category and those with some rain were divided into non-zero tertiles.

In order to understand the potential moderating effect of dog ownership and walking on weather–activity associations, interaction terms between the daily weather measurements and dog ownership categories were included in the models, and individual level covariates were adjusted for. The likelihood ratio test was conducted to examine whether the interaction terms achieved statistical significance. For the purpose of illustrating effect sizes, predicted activity levels and time spent sedentary by quartiles of environmental conditions and dog ownership status were computed based on the regression coefficient values. All analyses were conducted using Stata V.12.

## Results

Table 1 shows the sample characteristics by dog ownership status. Among the 3123 participants, the median age was 69.5 years (SD 7.6) with a range from 49 to 91 years, and 57% were female. Nearly 20% (n=573) reported having a dog in their household, with dog ownership declining with increasing age. Two-thirds of dog owners reported walking their dogs at least once a day with just 6% stating that they did not walk their dogs. Those reporting good health were more likely to be dog owners and to walk their dogs.

**Table 1:** Distributions of demographic factors and health status in the study sample

	Dog owner and dog walking	Dog owner but no dog walking	Non-dog owner	Total
N	383	190	2550	3123
Age group				
<65	105 (27.4)	64 (33.7)	677 (26.5)	846 (27.1)
65–69	99 (25.8)	53 (27.9)	546 (21.4)	698 (22.4)
70–74	90 (23.5)	31 (16.3)	544 (21.3)	665 (21.3)
75–79	57 (14.9)	25 (13.2)	453 (17.8)	535 (17.1)
80+	32 (8.4)	17 (8.9)	330 (12.9)	379 (12.1)
Gender				
Male	165 (43.1)	70 (36.8)	1113 (43.6)	1348 (43.2)
Female	218 (56.9)	120 (63.2)	1437 (56.4)	1775 (56.8)
Education				
Degree	54 (14.1)	26 (13.7)	457 (17.9)	537 (17.2)
A level	172 (45.0)	83 (43.7)	1168 (45.8)	1423 (45.6)
O level	46 (12.0)	29 (15.3)	295 (11.6)	370 (11.8)
No education	110 (28.8)	52 (27.4)	630 (24.7)	792 (25.4)
Self-reported health				
Excellent/very good/good	322 (84.1)	142 (74.7)	2144 (84.1)	2608 (83.5)
Fair/poor	61 (15.9)	48 (25.3)	406 (15.9)	515 (16.5)

### Physical activity, dog ownership and weather conditions

The 3123 participants provided a total of 21 235 valid days of accelerometer data. The mean daily physical activity counts per minute were 249.8 (SD 153.4). The sample spent an average of 667.1 minutes (SD 133.9) sedentary each day, which is equivalent to approximately 11 hours. Daily counts per minute were generally lower on days with higher precipitation (2.8 mm), lower temperature (<10.0°C) and shorter day length (<9.3 hours) while sedentary time was higher during these poorer conditions (table 2).

**Table 2:** The association between physical activity and weather conditions in the overall population (n=3123) adjusting for individual level factors (age, gender, education and self-rated health)

	Daily counts per minute	Sedentary time (min/day)
Precipitation (mm)		
0.0 (Ref)	–	–
0.2–0.6	−9.8 (−14.2 to −5.5)	4.2 (1.6 to 6.8)
0.6–2.6	−14.4 (−19.2 to −9.6)	7.8 (4.9 to 10.6)
2.8+	−24.9 (−29.6 to −20.2)	13.4 (10.7 to 16.3)
Max temperature (°C)		
19.2 (Ref)	–	–
14.3–19.1	−2.3 (−7.6 to 3.0)	5.5 (2.4 to 8.7)
10.0–14.2	−7.3 (−14.6 to 0.0)	7.6 (3.3 to 11.8)
<10	−16.7 (−25.1 to −8.3)	16.4 (11.4 to 21.3)
Day length (hour)		
14.90 (Ref)	–	–
11.80–14.85	−4.5 (−14.1 to 5.1)	5.7 (0.5 to 10.9)
9.28–11.75	−8.8 (−19.2 to 1.7)	8.8 (3.1 to 14.6)
<9.26	−9.6 (−21.0 to 1.8)	9.1 (2.8 to 15.4)

First-order autoregressive models included all individual (age, gender, education, self-rated health and disability) and weather factors.

Prior to adjustment, compared with dog owners who regularly walked their dogs, non-dog owners had lower daily counts per minute (−54.9; 95% CI: −66.2 to −43.7). Non-regular dog walkers had a similar level of physical activity to non-dog owners and showed lower daily counts per minute (−52.9; 95% CI: −71.1 to −34.7) than regular dog walkers.

Figure 1 (a–c) shows estimated daily counts per minute by different environmental conditions and dog ownership status adjusting for individual level factors. Across the whole sample, daily counts per minute were generally lower on days with higher precipitation. However, compared to dry days, regular dog walkers showed less decline (−37.0 cpm; 95% CI: −50.3 to −23.8) on wet days (2.8 mm precipitation) compared to non-dog owners (−80.0; 95% CI: −92.6 to −67.3) (figure 1a).

**Figure 1a:**
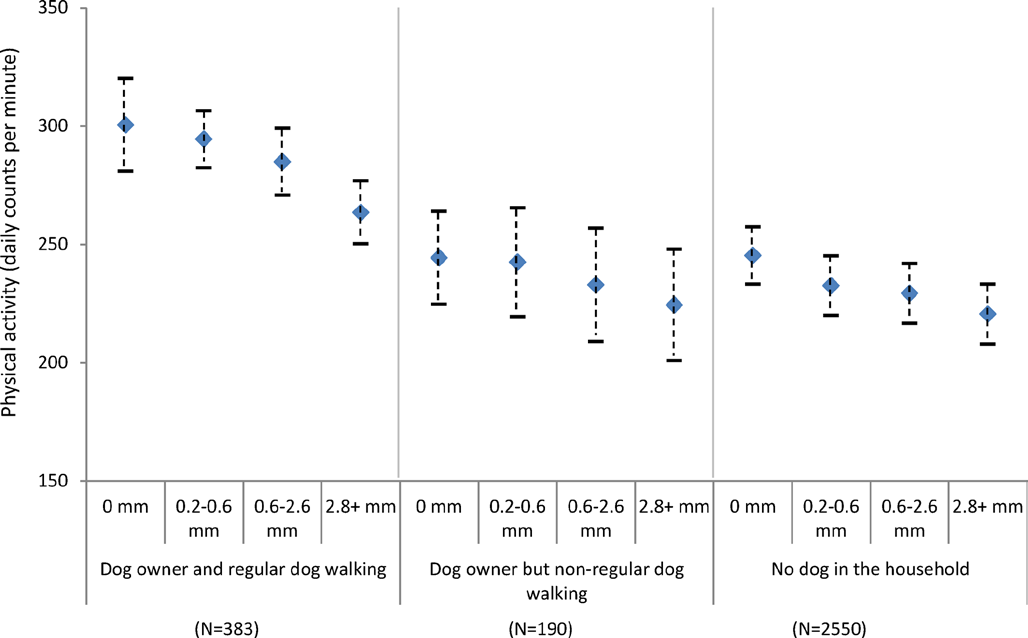
Estimated physical activity (daily counts per minute) by precipitation levels and dog ownership, adjusting for individual level factors (age, gender, education and self-rated health).

There was a decline in physical activity with decreasing maximum temperature in all groups except dog owners who did not regularly walk their dogs (figure 1b). However, even on the days with lower maximum temperature (<10.0°C), regular dog walkers were more active (275.1 cpm; 95% CI: 254.9 to 295.3) than non-regular dog walkers (242.6 cpm; 95% CI 270.1 to 215.2) or those who did not own a dog (249.6 cpm; 95% CI: 233.4 to 265.9) were on the days with the highest maximum temperature (19.2°C). Although there was a decline in physical activity with decreasing day length for all groups, even on the shortest days regular dog walkers were again more active (289.7 cpm; 95% CI: 262.9 to 316.5) than their non-dog owning (241.8 cpm; 95% CI: 208.4 to 275.1) or non-regular dog walking counterparts (249.8 cpm; 95% CI: 230.5 to 269.2) were on the longest days (figure 1c).

**Figure 1b:**
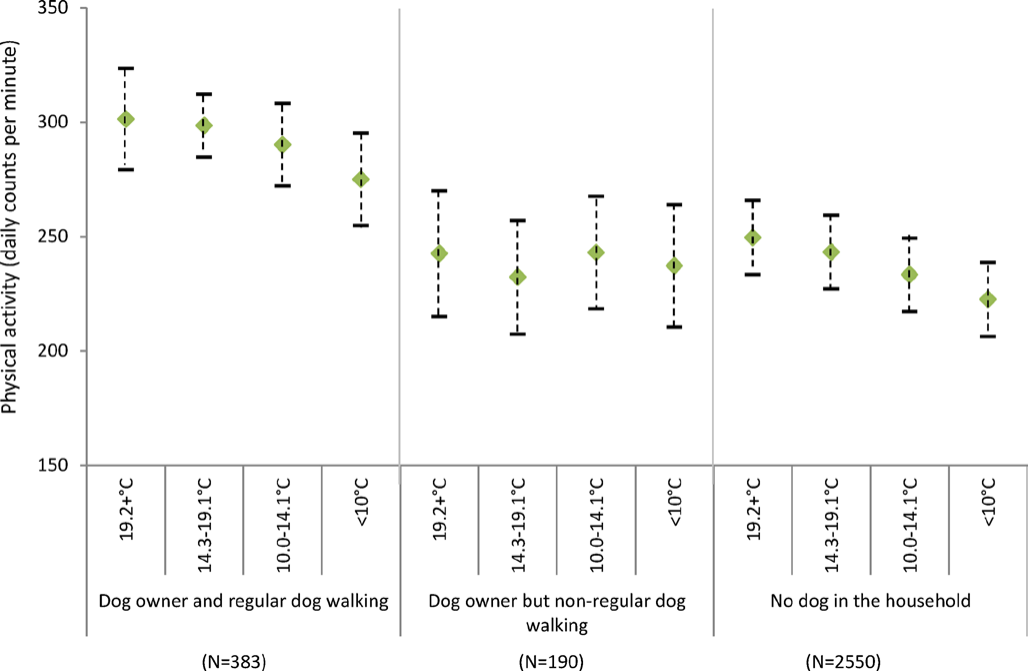
Estimated physical activity (daily counts per minute) by daily maximum temperature and dog ownership, adjusting for individual level factors (age, gender, education and self-rated health).

**Figure 1c:**
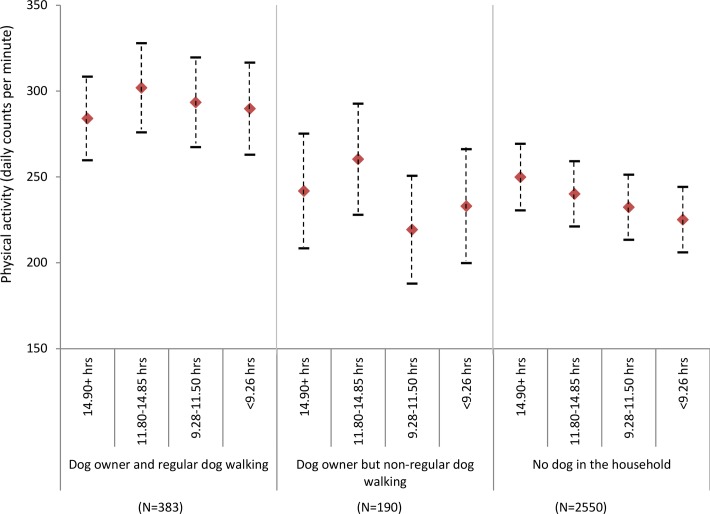
Estimated physical activity (daily counts per minute) by day length and dog ownership, adjusting for individual level factors (age, gender, education and self-rated health).

Figure 2 (a–c) shows adjusted time spent sedentary by different environmental conditions and dog ownership status. Overall, dog owners were less sedentary than those who did not own dogs, and this was particularly the case amongst those reporting regular dog walking. Although time spent sedentary was higher with poorer environmental conditions across all groups, dog walkers were most active in all conditions. For example, regular dog walkers recorded 632 (95% CI: 617.3 to 645.9) sedentary minutes on days with no precipitation compared to 648.6 (95% CI: 640.7 to 656.5) minutes in the wettest days. For non-dog owners, sedentary time ranged from 660.6 minutes (95% CI: 654.1 to 667.1) on dry days to 675.7 minutes (95% CI: 668.9 to 682.6) on the wettest days (figure 2a). For all three exposures, dog owners who regularly walked their dogs were generally less sedentary on days with the worst conditions than non-dog owners were on days with the best conditions (figure 2a-c).

**Figure 2a:**
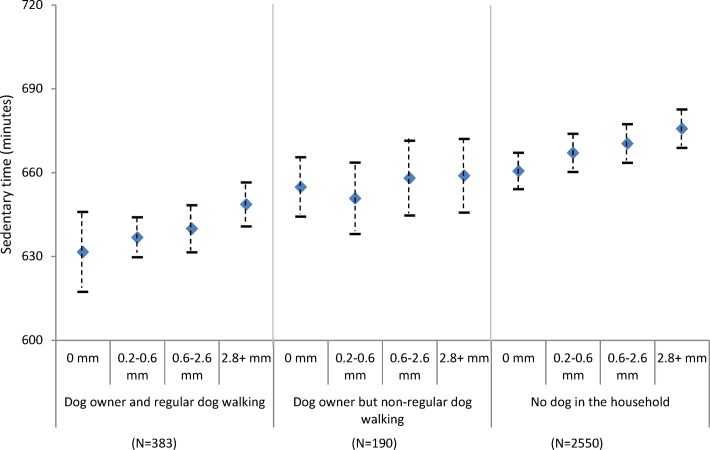
Estimated sedentary time (minutes per day) by precipitation levels and dog ownership, adjusting for individual level factors (age, gender, education and self-rated health).

**Figure 2b:**
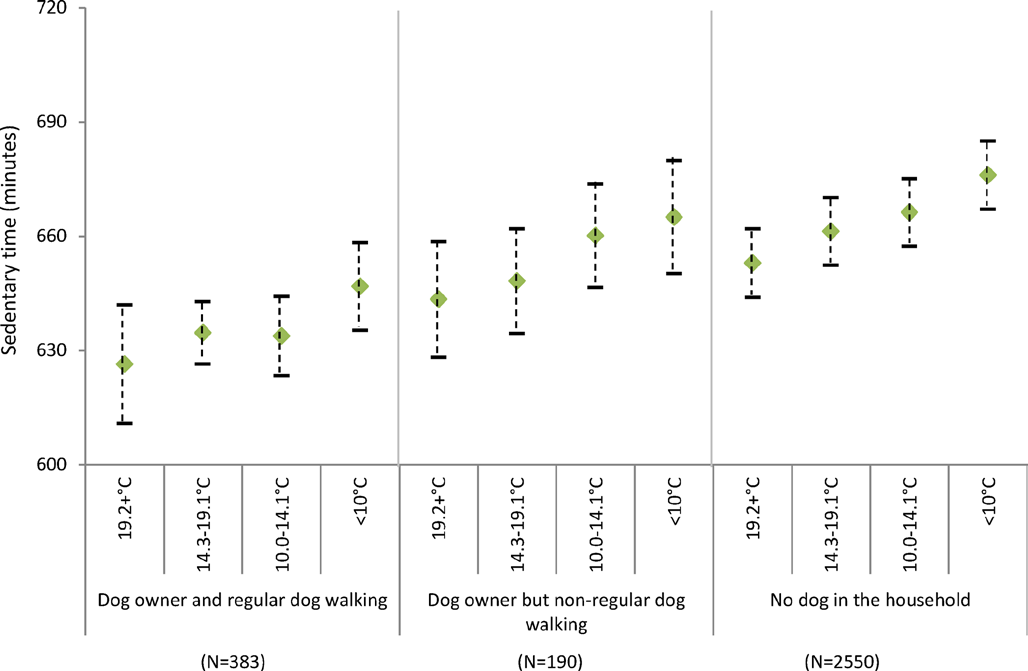
Estimated sedentary time (minutes per day) by daily maximum temperature and dog ownership, adjusting for individual level factors (age, gender, education and self-rated health).

**Figure 2c:**
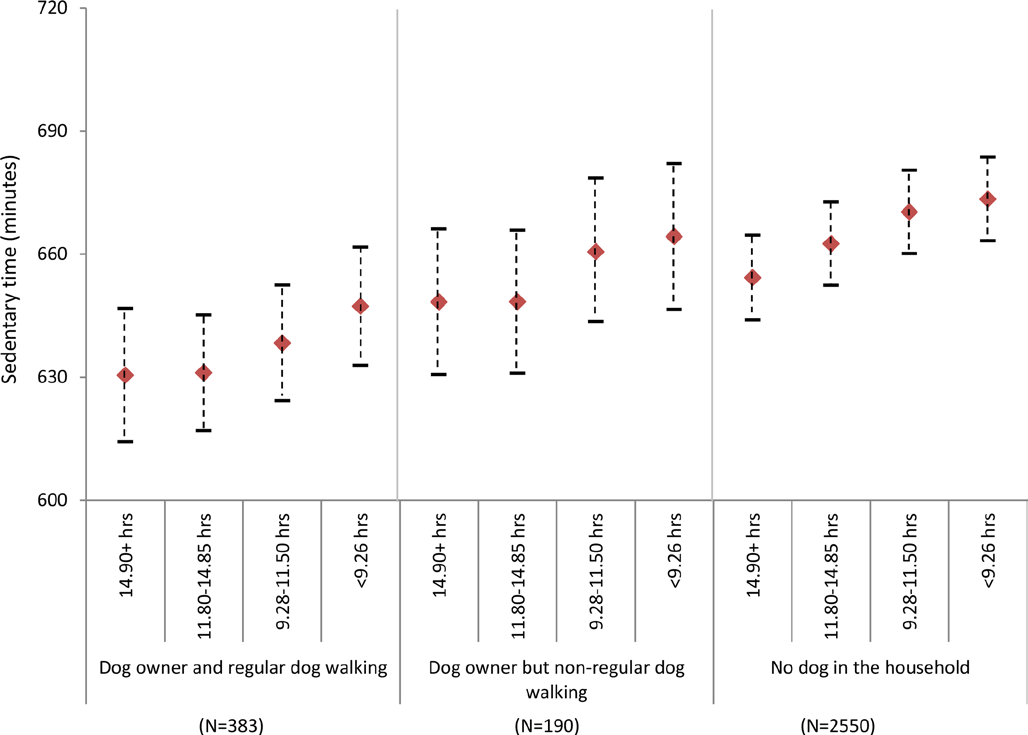
Estimated sedentary time (minutes per day) by day length and dog ownership, adjusting for individual level factors (age, gender, education and self-rated health).

## Discussion

### Main findings

Short day length, heavy rain and low temperature were associated with lower physical activity and more time spent sedentary in this sample of older adults, yet dog owners recorded higher activity levels and shorter sedentary time even in days with poor environmental conditions. In the shortest days, and those with lower temperatures and higher precipitation, regular dog walkers recorded physical activity levels that were typically 20% higher than non-dog owners and they spent around 30 min/day less sedentary. Indeed, the magnitude of disparities was such that dog owners who regularly walked their dogs were on average more active and less sedentary on days with the poorest conditions than non-dog owners were on the days with the best conditions.

### Strengths and limitations

This study was based on a large population-based cohort of older English adults with objective measures of physical activity and detailed questions on dog ownership and dog walking. Compared with existing studies, we were able to distinguish dog walking habits from dog ownership and further stratify dog ownership status based on this factor. Information on objectively measured daily levels of physical activity and sedentary behaviour was linked to objective measures of daily weather conditions recorded from the national meteorological network, and time-series models were fitted to account for the temporal autocorrelation. Data were recorded over the whole year, maximising the heterogeneity present in all of the exposure variables.

In terms of limitations, the cross-sectional nature of our analysis means we cannot rule out the possibility of reverse causality, where more active individuals are more likely to own dogs. We did not have information on daily dog walking habits and hence the direction of the association cannot be determined. Although some individual and environmental factors such as functional ability, mobility and environmental supportiveness for dog walking might influence physical activity as well as the likelihood of dog ownership and walking habits, they are unlikely to confound associations with weather conditions. The clear differences observed between dog owners who did and did not report regular walking suggest that our findings were unlikely to be biased from residual confounding with unmeasured factors. The EPIC Norfolk is a longitudinal community-based cohort and a small number of participants may have moved to institutionalised settings at the 3HC. However, the vast majority of participants remained community dwelling.^25^

The climate in East of England is less extreme compared with some other regions or countries, and the protective effect of dog ownership could thus differ in areas with greater seasonal disparities. The analyses only focused on those who reported provided information on dog ownership and around a quarter of the sample did not complete this part of the survey. While there may therefore be some selection bias, the prevalence of dog ownership in this sample was similar to that reported in another UK study.^31^

### Relationship with other studies

We found regular dog walkers had a higher level of physical activity and spent less time sedentary than their non-dog owning or non-regular dog walking counterparts regardless of day length and weather conditions. The findings support indications from two observational studies in Canada.^22 23^ A postal survey of 428 adults in Calgary showed that reported recreational walking time in dog owners was over twice as high as that reported by non-dog owners in the winter.^22^ An observational study of six public parks in Victoria, British Columbia, found that during the months of poor weather, numbers of individuals observed walking without dogs fell significantly, but there was no significant reduction in the number of dog walkers’ visits.^23^ In other work, dog ownership has been found to be a strong source of motivation, companionship and social support.^32^ There is evidence of potential drivers of the observation in qualitative research that has described how older adults report being more motivated to get out of doors with their dogs even on days with poor weather.^19^ Using objective measures of physical activity and weather conditions, our findings support those from these studies.

The fact that we did not evaluate an intervention means effect sizes cannot be compared. However, a meta-analysis summarising results from 15 trails in older adults in primary care suggested only small effects of exercise referral interventions on self-reported physical activity at 12 months.^2^ Our study shows up to 22% higher activity levels in dog owners than non-dog owners in the poorest environmental conditions. This indicates that dog ownership, in particular dog walking, has the potential to be an effective component of physical activity promotion in this population.

### Implications for clinicians and policymakers

Our findings suggest that dog ownership and walking may have considerable potential to support the maintenance of physical activity in older adults and could form part of exercise on prescription schemes. Nevertheless, dog ownership decreased with age in our sample, which highlights concerns regarding the appropriateness of encouraging dog ownership; while older adults might have more free time, declines in health status or housing conditions can limit the ability of individuals to care for dogs in the household.^31^ In cases where dog ownership is not possible but where the functional status allows, dog walking opportunities for older adults who do not own a dog could be organised by local community organisations or charities, and dog walking groups may provide wider well-being benefits associated with increased social contact.^32^ Links might be made, for example, with groups such as the ‘Borrow My Doggy,’ a nationwide network in UK^33^ which provides regular group walks for non-dog owners looking for the opportunity to walk one. As these opportunities may confer the broader group-based benefits to health and well-being associated with walking groups^34^ they should be explored with patients where appropriate.

Recent reviews have suggested environmental supportiveness for dog walking and human–animal interactions are likely important components of physical activity promotion efforts that might make use of dog walking opportunities.^21 35^ Public health interventions may therefore benefit from additional consideration of social and physical environmental factors which support older adults to walk their dogs in neighbourhoods. Some possible directions include pet-friendly policies in retirement communities^11^ and environmental modifications on dog-supportive features such as creating off-leash areas and dog walking trails in parks and green space.^11 36^

### Unanswered questions and future research

As it may be unethical to allocate dogs in randomised trials, before and after approaches are likely to be fruitful to examine if changes in physical activity follow the initiation of dog ownership. For example, a longitudinal study of new home owners in Perth, Australia, compared changes in recreational walking over 12 months between non-dog owners and those who owned a dog only at follow-up.^37^ The results show that new dog owners had a greater increase in recreational walking minutes per week compared with non-dog owners. A recent natural experiment study in Calgary, Canada, investigated changes in visitor profiles and activities before and after dog-supportive modification was made to parks.^36^ The findings suggest that accommodating off-leash areas in parks has the potential to modify park use patterns and activities but may not increase visits among dog walkers in the short term. Work is also needed to understand how dog walking might practically be incorporated into exercise referral schemes. Outside primary care, social prescribing^38^ may offer a potentially attractive opportunity for dog walking and interactions with dogs in a supportive environment. Interventions in physical activity promotion have been typically based on the Health Belief Model or Social Cognitive Theory^39^ and have therefore focused on addressing self-efficacy, perceived benefits and barriers.^40 41^ Our findings hint at the important additional role of extrinsic motivation, in this case the need for the dog to be exercised even in poor weather.

**What is already known on this subject**

- Dog ownership has been suggested as a potential way to encourage physical activity in older adults.
- Qualitative research has suggested that dog ownership can be a source of motivation and companionship which supports older people to walk outside on days with poor weather.
- Previous observational studies suggested that dog owners were more resilient to inclement seasonal conditions but none included objective measures for physical activity and daily weather.

**What this study adds**

- In our sample of older adults, dog owners who walked their dogs were more active and less sedentary on days with the poorest conditions than non-dog owners were on days with the best conditions.
- In the poorest conditions those who walked their dogs had 20% higher activity levels than non-dog owners and spent 30 min/day less sedentary.
- Encouraging dog ownership or dog walking where appropriate might form a potent component of interventions in primary care to support physical activity in older patients.
